# Diagnostic Sensitivity of the McMaster Technique for Detecting Intestinal Parasites in Human Populations: A Systematic Review and Meta-Analysis

**DOI:** 10.3390/tropicalmed11090249

**Published:** 2026-09-01

**Authors:** Jurairat Jongthawin, Aongart Mahittikorn, Kinley Wangdi, Frederick Ramirez Masangkay, Manas Kotepui

**Affiliations:** 1 Faculty of Medicine, Mahasarakham University, Maha Sarakham 44000, Thailand; jurairat.j@msu.ac.th; 2 Department of Protozoology, Faculty of Tropical Medicine, Mahidol University, Bangkok 10400, Thailand; aongart.mah@mahidol.ac.th; 3 HEAL Global Research Center, Health Research Institute, Faculty of Health, University of Canberra, Bruce, ACT 2617, Australia; kinley.wangdi@canberra.edu.au; 4 National Centre for Epidemiology and Population Health, College of Law, Governance and Policy, Australian National University, Acton, ACT 2601, Australia; 5 Department of Medical Technology, Faculty of Pharmacy, University of Santo Tomas, Manila 1008, Philippines; frmasangkay@ust.edu.ph; 6 Research Center for the Natural and Applied Sciences, University of Santo Tomas, Manila 1008, Philippines; 7 Medical Technology Program, Faculty of Science, Nakhon Phanom University, Nakhon Phanom 48000, Thailand

**Keywords:** McMaster, flotation techniques, intestinal parasites, soil-transmitted helminths, STHs, *Ascaris lumbricoides*, *Trichuris trichiura*, hookworms

## Abstract

Intestinal parasitic infections, particularly soil-transmitted helminths (STHs), remain a major public health concern in low- and middle-income countries. As control programs reduce infection intensity, accurate diagnosis becomes increasingly challenging. The McMaster technique has been proposed as an alternative diagnostic method for detecting human intestinal parasites; however, its diagnostic performance in human populations remains unclear. This study aimed to evaluate the diagnostic sensitivity of the McMaster technique for detecting intestinal parasites, particularly STHs. A systematic review and meta-analysis were conducted in accordance with the PRISMA guidelines and registered in PROSPERO (CRD420261298952). Electronic databases (PubMed, Scopus, Web of Science, Ovid, Nursing & Allied Health Premium, and Google Scholar) were searched up to January 2026. Studies assessing the McMaster technique in human populations and reporting sufficient data to calculate sensitivity were included. A random-effects model was used to estimate pooled sensitivity, with subgroup analyses by parasite species, geographic region, and population characteristics. Risk of bias was assessed using the the Quality Assessment of Diagnostic Accuracy Studies-2 (QUADAS-2) tool. Among the 1514 records identified through database searching, 10 studies were included in the final analysis. The pooled sensitivity of the McMaster technique was 73% (95% confidence interval [CI]: 67–78%; *I*^2^ = 91.0%) for detecting any intestinal parasites and 72% (95% CI: 65–78%; *I*^2^ = 93.5%) for STHs. Species-specific sensitivities were 67% (95% CI: 61–72%; *I*^2^ = 80.7%) for *Ascaris lumbricoides*, 79% (95% CI: 57–91%; *I*^2^ = 96.9%) for *Trichuris trichiura*, and 71% (95% CI: 61–80%; *I*^2^ = 89.9%) for hookworms. The McMaster technique demonstrates moderate sensitivity for detecting intestinal parasites in humans. While it offers practical advantages for field applications, its reduced sensitivity in low-intensity infections limits its utility as a standalone diagnostic tool in control and elimination settings. Further research should focus on methodological standardization and integration with more sensitive diagnostic approaches.

## 1. Introduction

Intestinal parasitic infections, especially soil-transmitted helminths (STHs), continue to represent a significant global public health challenge, impacting an estimated 1.5 billion individuals globally, with the majority residing in low- and middle-income nations [1]. These infections are associated with significant morbidity, including malnutrition, iron-deficiency anemia, and impaired physical and cognitive development, especially among children and other vulnerable populations [2]. Over the past two decades, widespread mass drug administration (MDA) programs have clearly reduced the prevalence and intensity of STH infections in many endemic regions [3]. However, as transmission rates decline, infections are increasingly characterized by low parasite burdens, thus posing substantial challenges for accurate diagnosis and surveillance activities [4].

Reliable and sensitive diagnostic tools are essential for monitoring control programs, evaluating treatment effectiveness, and identifying ongoing transmission. The Kato–Katz (KK) method has long been recommended by the World Health Organization (WHO) as one of the standard copromicroscopic methods for diagnosing STH infections in epidemiological surveys and monitoring preventive chemotherapy programs because of its simplicity, low cost, and ability to estimate infection intensity [5,6,7]. However, the Kato–Katz method has key limitations, particularly its reduced sensitivity for low-intensity infections and the quick breakdown of hookworm eggs. This requires slides to be examined within 30–60 min of preparation, which limits their use in field settings [8].

The McMaster technique is a flotation-based quantitative fecal egg counting method originally developed for veterinary parasitology [9], where it has been widely used to estimate parasite burden as eggs per gram (EPG), monitor infection dynamics, and evaluate anthelmintic efficacy because of its simplicity, rapidity, low cost, and standardized approach [10]. In the conventional McMaster procedure, approximately 1 g of feces is homogenized in 14 mL of flotation solution [9], and a measured volume of the suspension is examined in a specialized counting chamber. This protocol typically provides an analytical sensitivity (limit of detection) of approximately 100 eggs per gram [11], making the method less sensitive for detecting light-intensity infections. Compared with the Kato–Katz method, the McMaster technique is less dependent on immediate microscopic examination after stool preparation and may therefore offer logistical advantages for field-based sample processing, particularly for hookworm detection [10]. Nevertheless, its analytical sensitivity is limited by the dilution inherent to the method, making it less suitable for detecting low-intensity infections, including those commonly observed following preventive chemotherapy or in low-transmission settings [12]. These methodological variations reflect attempts to optimize the technique for human diagnosis but have resulted in substantial heterogeneity across studies, potentially affecting analytical sensitivity and complicating direct comparisons of diagnostic performance.

Notwithstanding these benefits, the diagnostic precision of the McMaster technique in human populations has not been definitively established. Its analytical sensitivity is subject to dilution factors inherent to the flotation process, potentially diminishing its ability to detect low-intensity infections and increasing the likelihood of false-negative outcomes [10,13]. Prior investigations have documented variation in sensitivity across different geographical areas, study cohorts, and parasite species; furthermore, evidence indicates that its efficacy may be lower than that of Kato–Katz in identifying low-intensity infections [4,10]. Presently, a clear consensus regarding its performance relative to other established copromicroscopic diagnostic methods, including Mini-FLOTAC and concentration techniques, is lacking [4,13].

Consequently, a thorough synthesis of existing data is necessary to elucidate the diagnostic value of the McMaster technique in human parasitology. This systematic review and meta-analysis aimed to assess the diagnostic sensitivity of the McMaster method against established composite reference standards for identifying intestinal parasites in human subjects. More specifically, this investigation intended to calculate the pooled sensitivity of the McMaster technique and to examine potential sources of heterogeneity, encompassing geographical location, participant demographics, and the specific parasite species involved. The findings will help clarify the diagnostic performance and limitations of the McMaster technique for detecting intestinal parasites in human populations.

## 2. Methods

### 2.1. Protocol and Registration

The present systematic review and meta-analysis were conducted in accordance with the Preferred Reporting Items for Systematic Reviews and Meta-Analyses (PRISMA) guidelines. The study protocol was registered with the International Prospective Register of Systematic Reviews (PROSPERO) under the registration number CRD420261298952.

### 2.2. Definitions

For this systematic review and meta-analysis, the term “composite reference standard” refers to the comparator diagnostic method(s) used in each included study (e.g., Kato–Katz, formalin–ether concentration technique, Mini-FLOTAC, or other accepted copromicroscopic methods), against which the McMaster technique’s diagnostic performance was evaluated. 

### 2.3. Review Framework

This systematic review was developed using a diagnostic test accuracy (DTA) framework based on the population, index test, reference standard, and diagnosis (PIRD) approach [14,15]. The population of interest was human participants of all age groups, including community members, schoolchildren, and clinical patients at risk of intestinal parasitic infections. The index test was the McMaster technique, applied to stool samples to detect and quantify parasites. The reference standard consisted of Kato–Katz, Mini-FLOTAC, formol–ether concentration, and other accepted copromicroscopic methods, used either alone or in combination. The target condition was the presence of intestinal parasitic infections, particularly STHs (*A. lumbricoides*, *T. trichiura*, and hookworms), with the primary outcome being the diagnostic sensitivity of the McMaster technique.

### 2.4. Information Sources and Search Strategy

A comprehensive literature search was conducted across six electronic databases, including PubMed, Scopus, Ovid, Nursing & Allied Health Premium, Web of Science, and Google Scholar, from database inception to 30 January 2026. Google Scholar was searched separately on 2 February 2026. The search strategy combined controlled vocabulary (e.g., Medical Subject Headings [MeSH] in PubMed) with free-text keywords related to three main concepts: McMaster, intestinal parasites, and human populations. Boolean operators (AND/OR), truncation, phrase searching, and database-specific indexing terms were applied as appropriate for each database. The search strategies were adapted to accommodate differences in database syntax and indexing while maintaining the same conceptual framework. For Google Scholar, the first 200 records, sorted by relevance, were screened due to the large number of retrieved results [16]. The complete search strategies, including the exact search strings used for each database, are provided to ensure reproducibility (Table S1).

### 2.5. Eligibility Criteria

Studies were considered eligible if they met the following criteria: (i) involved human participants of any age group residing in endemic or non-endemic settings; (ii) used the McMaster technique in detecting intestinal parasites; (iii) included at least one established reference standard (e.g., Kato–Katz, Mini-FLOTAC, or concentration techniques) or a composite reference standard as a comparator; (iv) provided sufficient data to calculate diagnostic sensitivity (i.e., true positives and false negatives); and (v) employed a cross-sectional, cohort, or diagnostic accuracy study design. Studies were excluded if they were conducted exclusively in animals, lacked sufficient data to calculate sensitivity, were reviews, editorials, or case reports, or used only molecular methods without a composite reference standard comparison.

### 2.6. Study Selection and Data Extraction

All identified records were imported into the EndNote version 21.0 (Philadelphia, PA, USA) reference management software, and duplicates were removed. Two independent reviewers (JJ, MK) screened titles and abstracts for relevance, followed by full-text assessment of potentially eligible studies. Inter-reviewer agreement for study selection was assessed using Cohen’s kappa and was substantial (κ = 0.73). Disagreements between reviewers were resolved through discussion or consultation with a third reviewer (AM).

A standardized data extraction form was used. The following information was collected: study characteristics (author, year, country, region), participant characteristics (age group, setting: community, school, or clinical), parasite species investigated (e.g., *A. lumbricoides*, *T. trichiura*, hookworms), diagnostic methods used (index and reference tests), outcome data (true positives, false negatives, sample size). Data extraction was performed independently by two reviewers (JJ, MK), with discrepancies resolved by consensus.

### 2.7. Quality Assessment

The methodological quality of included studies was assessed using the Quality Assessment of Diagnostic Accuracy Studies-2 (QUADAS-2) tool [17]. This tool evaluates four domains, including patient selection, index test, reference standard, and flow and timing. Each domain was assessed for risk of bias and applicability concerns. Quality assessment was performed independently by two reviewers (JJ, MK), with discrepancies resolved by consensus. Risk-of-bias assessments were visualized using the robvis web application [18].

### 2.8. Data Synthesis and Statistical Analysis

The primary outcome was the pooled sensitivity of the McMaster technique for detecting intestinal parasites. Because most included studies did not report specificity or complete 2 × 2 contingency table data (true positives, false positives, false negatives, and true negatives), hierarchical DTA models, such as the bivariate random-effects model or the hierarchical summary receiver operating characteristic (HSROC) model, could not be applied. Therefore, a meta-analysis of proportions using a random-effects model was performed to estimate pooled sensitivity while accounting for between-study heterogeneity. Sensitivity estimates were pooled across all analyses and stratified by parasite group (any intestinal parasites and STHs), STH species (*A. lumbricoides*, *T. trichiura*, and hookworms), geographic region, and participant characteristics (community-, school-, and clinical-based populations). Where sufficient data were available, subgroup analyses were also conducted according to the reference standard (e.g., Kato–Katz, Mini-FLOTAC, formalin–ether concentration, or composite reference standards) to explore the influence of different comparator methods on pooled sensitivity estimates. Between-study heterogeneity was assessed using the *I*^2^ statistics.

Potential sources of heterogeneity were explored through predefined subgroup analyses based on parasite species, geographic region, participant characteristics, and reference standard. Infection intensity was also considered a clinically important source of heterogeneity because the McMaster technique’s analytical sensitivity depends on fecal egg counts. However, stratified analyses by infection intensity were not performed because the included studies did not consistently report egg counts or use comparable EPG categories, precluding meaningful quantitative synthesis. Because the number of studies within several subgroups was limited and methodological variables (e.g., flotation solution, egg counting protocol, infection intensity, endemicity, and age group) were incompletely reported, meta-regression was not performed, as such analyses would have been statistically underpowered and potentially unreliable. Publication bias was assessed using funnel plots, Egger’s regression test, and the trim-and-fill method. Given the limited number of included studies, these analyses were considered exploratory and interpreted with caution. All statistical analyses were performed using RStudio (Version 2024.04.2+764) [19], with statistical significance set at *p* < 0.05.

## 3. Results

### 3.1. Study Selection

**Figure 1:**
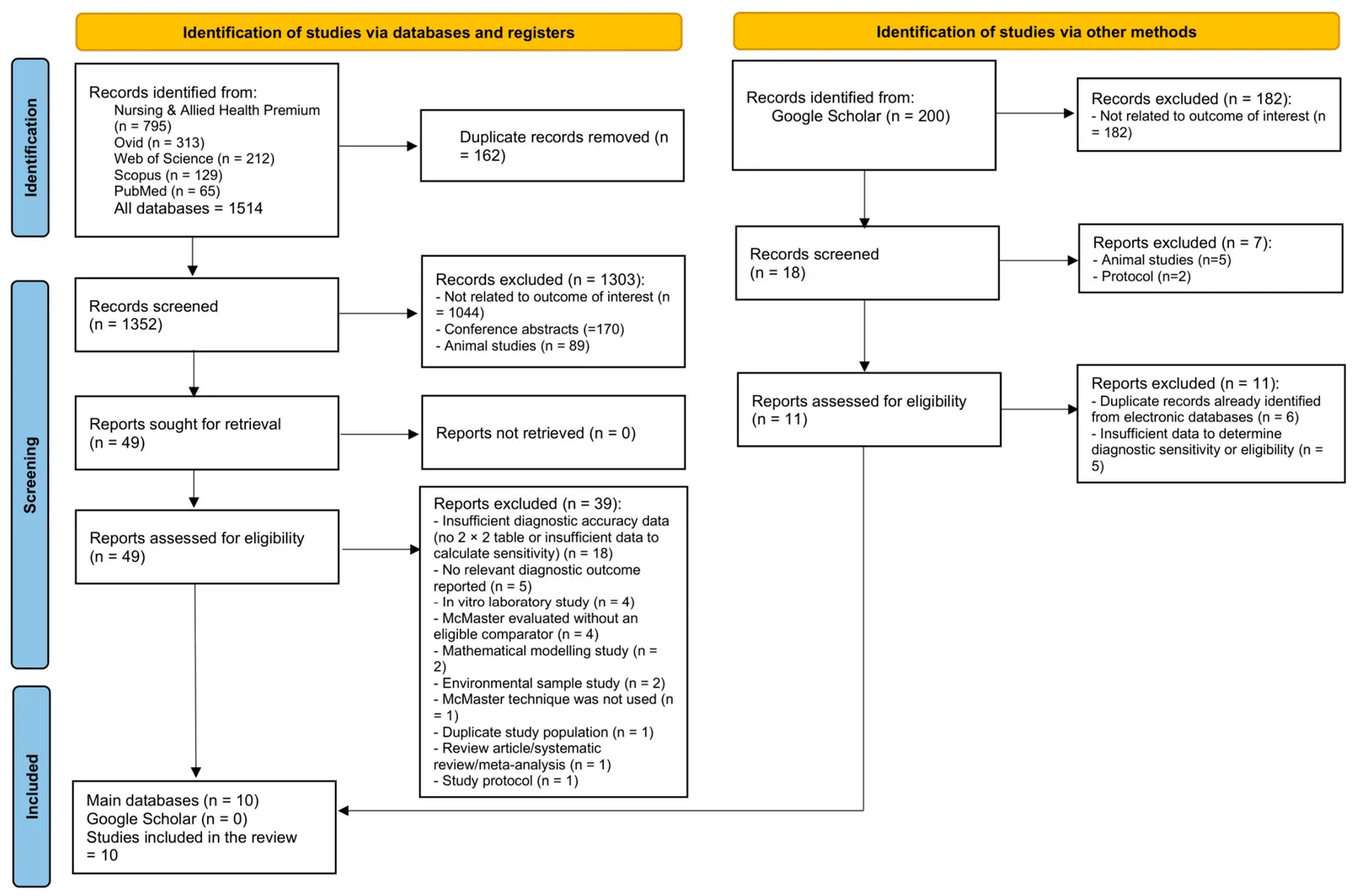
Study selection diagram (PRISMA diagram).

A total of 1514 records were identified through database searches, with an additional 200 identified via Google Scholar. After removing 162 duplicates, 1352 records were screened by title and abstract; 1303 were excluded for failing to meet the inclusion criteria. Forty-nine full-text articles were assessed for eligibility, and 39 were excluded for reasons including lack of 2 × 2 diagnostic data, absence of relevant outcome data, in vitro or animal studies, use of the McMaster technique without a comparator, and other methodological limitations. From Google Scholar, 18 records were screened after initial filtering, with 11 full-text articles assessed; all were excluded due to duplication or insufficient data. Finally, 10 studies [10,20,21,22,23,24,25,26,27,28] met the inclusion criteria and were included in the final systematic review and meta-analysis (Figure 1).

### 3.2. Characteristics of Included Studies

Included studies were published between 2011 and 2025. Half of the studies (5/10, 50%) were cross-sectional in design [20,23,24,27,28], while the remainder included diagnostic accuracy studies [10,21,22,25], and one retrospective study [26]. Five studies (50%) were conducted in African countries [20,21,23,24,28] (Ethiopia [20,23,24,28] and Tanzania [21]), while the rest were from Asia (Lao PDR) [25], South America (Argentina [22,26], Brazil [27]), and one multi-continent study [10]. The majority of studies (7/10, 70%) enrolled schoolchildren [10,20,21,22,23,24,27], whereas the remaining studies included villagers [25], adult patients attending health facilities [28], and outpatients participating in public health surveys and interventions [26]. All included studies (10/10, 100%) investigated intestinal parasitic infections and STHs [10,20,21,22,23,24,25,26,27,28]. Species-specific analyses were reported for *A. lumbricoides* (9/10, 90%) [10,20,21,22,23,24,25,26,27], *T. trichiura* (7/10, 70%) [10,20,21,23,24,25,27], and hookworms (9/10, 90%) [10,20,21,23,24,25,26,27,28]. Details of the included studies are presented in Table S2.

### 3.3. Risk of Bias

**Figure 2:**
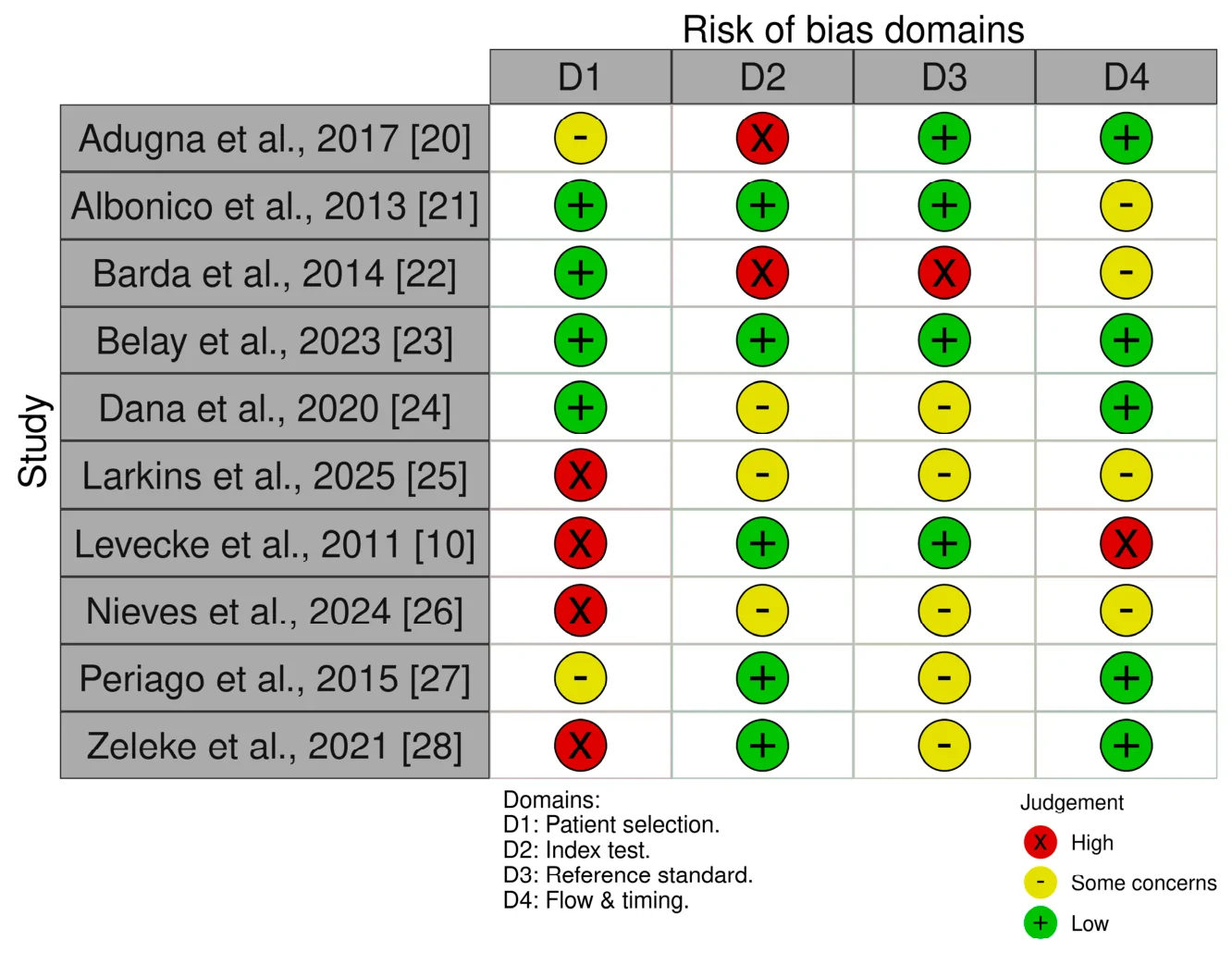
Traffic-light plot showing the risk of bias among included studies across the four QUADAS-2 domains [10,20,21,22,23,24,25,26,27,28].

Most studies showed low risk of bias in the reference standard and flow and timing domains, as appropriate diagnostic methods were used, and most of the participants were included in the analyses [20,21,23,24,28]. However, concerns were identified in the patient selection domain due to non-random sampling in several studies [10,25,26,28], and in the index test domain, where blinding was often unclear or not performed [20,22,24,25,26]. Overall, the main sources of potential bias were related to participant selection and lack of blinding in test interpretation (Figure 2).

### 3.4. Sensitivity of the McMaster Technique for Detecting Intestinal Parasites

The pooled sensitivity of the McMaster for detecting intestinal parasites is shown in Table 1. Overall, the McMaster method showed a pooled sensitivity of 73% (95% confidence interval [CI]: 67–78%, 10 studies) for detecting any intestinal parasites when compared with the reference standard used in each study. Subgroup analyses by geographical region and participant characteristics showed variation in diagnostic sensitivity. Among regions represented by multiple studies, the pooled sensitivity was 75% in Africa (5 studies) and 66% in South America (3 studies). Only one study was available from Asia; therefore, the reported sensitivity of 77% reflects the findings of that individual study rather than a pooled meta-analytic estimate and should be interpreted with caution. Among the available studies, reported country-specific sensitivity estimates ranged from 82% in Tanzania to 63% in Argentina. Similarly, the villager subgroup was represented by only one study, and the reported sensitivity of 77% should be interpreted as an estimate from that study rather than a pooled summary. In comparison, pooled sensitivities were 73% among schoolchildren (7 studies) and 65–69% among patients in clinical settings (2 studies).

### 3.5. Sensitivity of the McMaster Technique for Detecting Any STHs

For detecting any STHs, the pooled sensitivity was 72% (95% CI: 65–78%; 10 studies). Species-specific analyses yielded pooled sensitivities of 67% (95% CI: 61–72%; 9 studies) for *A. lumbricoides*, 79% (95% CI: 57–91%; 7 studies) for *T. trichiura*, and 71% (95% CI: 61–80%; 9 studies) for hookworms.

Regional subgroup analyses demonstrated pooled sensitivities of 73% in Africa (5 studies) and 66% in South America (3 studies). Because only one study was available from Asia, the reported sensitivity of 77% reflects the result of that single study rather than a pooled estimate and should be interpreted with caution. Likewise, the villager subgroup included only one study, whereas pooled sensitivities were 71% among schoolchildren (7 studies) and 65–69% among clinical populations (2 studies).

### 3.6. Sensitivity of the McMaster Technique for Detecting Species-Specific STHs

For *A. lumbricoides*, pooled sensitivity was generally consistent across regions represented by multiple studies, ranging from 64% to 68%, with country-level estimates ranging from 74% in Tanzania to 60% in Argentina. The reported sensitivity for hospital-based populations (62%) was derived from a single study and should therefore be interpreted with caution.

For *T. trichiura*, marked regional variation was observed. However, the estimates for both Asia (94%; 95% CI: 88–96%) and South America (47%; 95% CI: 21–73%) were each based on a single study and therefore represent individual study findings rather than pooled regional estimates. Similarly, the reported sensitivity among villagers (94%) was based on a single study, whereas the pooled sensitivity among schoolchildren (70%) was derived from six studies.

For hookworm detection, pooled sensitivity was 77% in Africa (5 studies) and 76% in South America (2 studies). The estimate of 62% for Asia was based on a single study and should be interpreted cautiously. Country-level estimates ranged from 81% in Brazil to 62% in Lao PDR. Likewise, the sensitivity estimate for villagers (62%) was derived from a single study, whereas the pooled sensitivity among schoolchildren (76%) was based on six studies.

### 3.7. Publication Bias and Sensitivity Analysis

Publication bias was assessed using funnel plots, Egger’s test, and Trim-and-Fill analysis. Although funnel plot asymmetry was observed for overall intestinal parasite detection, Egger’s test was not statistically significant (*p* > 0.05). Trim-and-Fill adjustment slightly reduced pooled sensitivity from 73% to 70% (95% CI: 62–77%) for any intestinal parasites and from 72% to 68% (95% CI: 59–76%) for STHs. The meta-analyses of species-specific STHs showed similar patterns, with adjusted sensitivities of 68% for *A. lumbricoides* and 67% for hookworm after imputation. Publication bias for *T. trichiura* was not assessed because fewer than 10 studies were included in the meta-analysis.

## 4. Discussion

This systematic review and meta-analysis evaluated the diagnostic sensitivity of the McMaster technique for detecting intestinal parasitic infections in human populations. Overall, the meta-analysis showed that the McMaster technique has moderate sensitivity, with pooled estimates of 73% for any intestinal parasites and 72% for STHs. These results suggested that while the technique is useful for detecting infections at the population level, it may miss a substantial proportion of cases, particularly in settings where infection intensity is low [13,29].

The observed sensitivity is consistent with the known methodological characteristics of the McMaster technique. As a flotation-based method with inherent dilution factors, its ability to detect low egg burdens is limited compared with thick smear techniques such as Kato–Katz or concentration-based approaches [10]. Nevertheless, the Kato–Katz technique remains the recommended standard for STH diagnosis [5]. However, like other copromicroscopic methods, its sensitivity decreases in low-intensity infections. This limitation is particularly relevant in the context of ongoing control programs, where reductions in infection intensity may further compromise diagnostic sensitivity. Therefore, the moderate sensitivity observed in this study suggests that prevalence estimates based solely on the McMaster technique should be interpreted cautiously. In addition, the McMaster technique may not be suitable for control settings where low-intensity infections are common, as its diagnostic performance tends to decline under these conditions [13]. Recent studies have shown that modified or concentration-based McMaster techniques may improve sensitivity compared with the conventional method for detecting *T. trichiura* and *Ascaris* spp. [12], supporting the continued evaluation of McMaster-based approaches in human diagnostics.

Subgroup analyses revealed substantial heterogeneity across geographical regions and study populations. Sensitivity tended to be higher in studies conducted in Africa and Asia compared with South America, although the number of studies in some subgroups was limited. This heterogeneity may reflect differences in infection intensity, laboratory procedures, or study design across regions [21,22]. Similarly, the higher sensitivity observed in community-based populations compared with clinical settings may be attributable to differences in parasite burden, sample-handling practices, or the limited number of studies evaluating diagnostic performance in these groups.

Species-specific analyses further demonstrated differences in sensitivity. Sensitivity for *A. lumbricoides* was relatively consistent across geographical regions, likely due to the higher egg output associated with this parasite [8], which facilitates detection even with less sensitive methods. Variability in egg excretion between stool samples is a well-recognized factor influencing diagnostic sensitivity [30]. In contrast, *T. trichiura* showed considerable variability in sensitivity, with markedly higher sensitivity in some settings than others. This may reflect differences in infection intensity or in the distribution of eggs in stool samples. Hookworm detection showed moderate sensitivity overall, with some regional variation; however, the McMaster technique may offer practical advantages for hookworm detection due to its reduced dependence on rapid slide reading compared with Kato–Katz.

The results of the meta-analysis should also be interpreted within the context of current WHO recommendations and recent advances in STH diagnostics. Current WHO guidance continues to recommend copromicroscopic methods, particularly Kato–Katz and other fecal egg-counting techniques, for epidemiological surveys and monitoring preventive chemotherapy programs because they are inexpensive, simple, and operationally feasible in resource-limited settings [6]. Within this framework, the McMaster technique remains a practical quantitative method for estimating infection intensity and monitoring programmatic outcomes. However, as STH control programs reduce transmission and infection intensity, the sensitivity of microscopy-based methods declines, particularly for light-intensity infections. Molecular methods, especially quantitative polymerase chain reaction (qPCR), have demonstrated substantially higher sensitivity and specificity and are increasingly regarded as valuable reference methods for surveillance and elimination programs [31]. This is consistent with the WHO Diagnostic Target Product Profiles, which prioritize more sensitive diagnostic tools as transmission declines and countries move toward elimination [31]. In addition, recent advances in digital microscopy and artificial intelligence (AI)-assisted image analysis [32,33,34] have shown promise for improving the accuracy, standardization, and throughput of parasite detection while reducing observer variability. Despite these advances, widespread implementation remains limited by cost, infrastructure requirements, technical expertise, and limited availability in many endemic regions. Therefore, the McMaster technique continues to have an important role in field-based epidemiological studies and routine monitoring, particularly where molecular diagnostics are not readily accessible. Future studies should evaluate how the McMaster technique performs alongside emerging molecular and AI-based diagnostic approaches and assess whether combinations of these methods can improve diagnostic accuracy across different transmission settings.

Some study limitations should be acknowledged. Several subgroup analyses, including those for Asia, villagers, and certain clinical populations, were based on a single study. Consequently, these values represent individual study findings rather than true pooled meta-analytic estimates and should be interpreted with caution. In addition, although the included studies represented three continents, they originated from a relatively small number of endemic countries, with several studies conducted in the same country. Therefore, the pooled estimates may not be fully generalizable to all endemic settings, particularly those with different epidemiological profiles, parasite transmission patterns, or diagnostic practices. Substantial between-study heterogeneity was observed across most pooled analyses (*I*^2^ > 90%), indicating considerable variability in the reported sensitivity estimates. Although subgroup analyses were performed according to parasite group, species, geographic region, and participant characteristics, substantial heterogeneity persisted, suggesting that additional methodological and epidemiological factors contributed to the observed variability. Potential sources of heterogeneity include differences in the reference standards used (e.g., Kato–Katz, Mini-FLOTAC, formalin–ether concentration, or composite reference standards), flotation solutions and McMaster protocols, stool preservation and processing procedures, infection intensity, endemicity, participant characteristics (e.g., age and clinical status), and study design. These factors are all known to influence the diagnostic sensitivity of copromicroscopic techniques. Among them, infection intensity is particularly important because the McMaster technique’s analytical sensitivity depends on fecal egg counts, which decline in low-intensity infections. However, the included studies did not consistently report EPG or use comparable infection-intensity categories, thereby precluding stratified analyses by infection intensity [10,35,36,37].

Although several included studies reported mean EPG values, these data were presented using different formats (e.g., means, mean ± standard deviation [SD], or species-specific values) and did not consistently classify infections according to standardized intensity categories (e.g., WHO light, moderate, and heavy infections). Consequently, quantitative subgroup analyses or meta-regression according to infection intensity were not feasible. Veterinary studies have similarly shown that the diagnostic sensitivity of the McMaster technique increases with increasing EPG and decreases at low egg counts, reflecting the analytical detection limit of the conventional method [35,36,37]. Although these findings provide important methodological support, they should not be directly extrapolated to human populations because of differences in host species, parasite biology, and laboratory procedures. Therefore, infection intensity could not be quantitatively evaluated in the present review, despite its recognized influence on diagnostic sensitivity. Future human diagnostic accuracy studies should report standardized EPG categories in accordance with WHO recommendations to facilitate infection-intensity-specific meta-analyses and improve comparisons across studies. Moreover, many methodological and epidemiological variables were reported inconsistently or were unavailable across studies, and the limited number of studies within individual subgroups precluded meaningful meta-regression or more detailed stratified analyses. Consequently, the individual contributions of these factors to the observed heterogeneity could not be quantified. Therefore, the pooled sensitivity estimates should be interpreted as average estimates across diverse study settings and populations with potentially different infection intensities, rather than as universal measures of diagnostic performance under specific transmission settings. Moreover, because only 10 studies were included in the meta-analysis, meta-regression was not performed, as it would likely yield unstable and unreliable estimates. Therefore, the pooled sensitivity estimates should be interpreted with caution.

The included studies used different reference standards, including Kato–Katz, Mini-FLOTAC, formalin–ether concentration techniques, and combinations of these methods used as composite reference standards, each with different diagnostic characteristics. This heterogeneity in comparator methods may have influenced the estimated sensitivity of the McMaster technique and contributed to the between-study variability observed in the pooled analyses. Although subgroup or sensitivity analyses by type of reference standard would have been valuable for evaluating the impact of comparator methods on the pooled estimates, such analyses were not feasible because only a small number of studies were available within each reference-standard category, and the reporting of comparator methods was inconsistent across studies. Consequently, the influence of reference-standard heterogeneity could not be evaluated quantitatively and should be considered an important limitation of this meta-analysis. Future studies that use standardized reference methods or sufficient data to enable reference-standard–specific subgroup analyses are needed to better characterize the diagnostic performance of the McMaster technique.

Reliance on copromicroscopic methods as reference standards, including composite reference standards when used, may also affect the accuracy of the pooled estimates [38]. Furthermore, most included studies did not report specificity or complete 2 × 2 contingency table data (true positives, false positives, false negatives, and true negatives). Consequently, hierarchical DTA models, such as the bivariate random-effects model or the HSROC model, could not be applied. Instead, pooled sensitivity was estimated using a random-effects meta-analysis of proportions. Although this approach is appropriate when only sensitivity data are available, it does not account for correlations between sensitivity and specificity or potential threshold effects; therefore, the pooled estimates should be interpreted with this limitation in mind. Although the risk-of-bias assessment indicated that most studies were of acceptable methodological quality, several studies raised concerns about patient selection and incomplete reporting of blinding procedures. Furthermore, while funnel plot asymmetry suggested potential publication bias, these findings should be interpreted with caution. Because only 10 studies were included and between-study heterogeneity was substantial, funnel plots and Egger’s regression had limited statistical power. Consequently, these analyses may have produced unreliable or misleading results. Therefore, the publication bias analyses should be considered exploratory rather than definitive, and the overall impact of these analyses on the pooled estimates remains uncertain. Finally, the absence of a true gold standard for STH diagnosis [13] may lead to an underestimation of the McMaster technique’s diagnostic accuracy.

## 5. Conclusions

The McMaster technique demonstrated moderate sensitivity for detecting intestinal parasites and STHs in human populations. While it remains a practical and cost-effective tool, particularly for field-based studies, its limitations in detecting low-intensity infections should be recognized. Future research should focus on standardizing methodologies, improving reporting quality, and evaluating the performance of the McMaster technique in combination with more sensitive diagnostic approaches, especially in low-transmission settings.

## Figures and Tables

**Table 1:** Sensitivity of the McMaster technique for detecting intestinal parasites compared with the reference standard.

Parasite Species	Subgroup Analysis	N	Pooled Sensitivity (95% CI) (%)	*I*^2^ (%)	Funnel Plot Asymmetry	*p*-Value of Egger’s Test	Trim and Fill Method	N. Studies (Subsets)	References
Any parasites	Overall	4889	73 (67–78)	91.0	Asymmetry	0.1057	70 (62–77) (2 added studies)	10 (23)	[10,20,21,22,23,24,25,26,27,28]
	**Continent**								
	Africa	1626	75 (63–85)	93.8	-	-	-	5 (7)	[20,21,23,24,28]
	South America	824	66 (58–74)	78.4	-	-	-	3 (7)	[22,26,27]
	Asia	1492	77 (60–89)	94.1	-	-	-	1 (6)	[25]
	Mixed (multi-continent)	947	76 (71–80)	63.7	-	-	-	1 (3)	[10]
	**Country**								
	Ethiopia	1150	70 (59–79)	93.8	-	-	-	4 (4)	[20,23,24,28]
	Tanzania	476	82 (56–94)	94.8	-	-	-	1 (4)	[21]
	Argentina	262	63 (57–69)	1.2	-	-	-	2 (4)	[22,26]
	Lao PDR	1492	77 (60–89)	94.1	-	-	-	1 (6)	[25]
	Brazil	562	70 (51–83)	86.3	-	-	-	1 (3)	[27]
	Mixed (multi-country)	947	76 (71–80)	63.7	-	-	-	1 (3)	[10]
	**Participants**								
	Schoolchildren	2966	73 (66–79)	90.2	-	-	-	7 (14)	[10,20,21,22,23,24,27]
	Villagers	1492	77 (60–89)	94.1	-	-	-	1 (6)	[25]
	Hospital patients/villagers	185	65 (57–73)	22.8	-	-	-	1 (2)	[26]
	Adult patients visiting health institutions	246	69 (63–74)	NA	-	-	-	1 (1)	[28]
Any STHs	Overall	4978	72 (65–78)	93.5	Asymmetry	0.2144	68 (59–76) (3 added studies)	10 (25)	[10,20,21,22,23,24,25,26,27,28]
	**Continent**								
	Africa	1715	73 (57–85)	96.4	-	-	-	5 (9)	[20,21,23,24,28]
	South America	824	66 (58–74)	78.4	-	-	-	3 (7)	[22,26,27]
	Asia	1492	77 (60–89)	94.1	-	-	-	1 (6)	[25]
	Mixed (multi-continent)	947	76 (71–80)	63.7	-	-	-	1 (3)	[10]
	**Country**								
	Ethiopia	1239	67 (46–83)	96.9	-	-	-	4 (6)	[20,23,24,28]
	Tanzania	476	82 (56–94)	94.8	-	-	-	1 (3)	[21]
	Argentina	262	63 (57–69)	1.2	-	-	-	2 (4)	[22,26]
	Lao PDR	1492	77 (60–89)	94.1	-	-	-	1 (6)	[25]
	Brazil	562	70 (51–83)	86.3	-	-	-	1 (3)	[27]
	Mixed (multi-country)	947	76 (71–80)	63.7	-	-	-	1 (3)	[10]
	**Participants**								
	Schoolchildren	3055	71 (62–79)	94.4	-	-	-	7 (16)	[10,20,21,22,23,24,27]
	Villagers	1492	77 (60–89)	94.1	-	-	-	1 (6)	[25]
	Hospital patients/villagers	185	65 (57–73)	22.8	-	-	-	1 (2)	[26]
	Adult patients visiting health institutions	246	69 (63–74)	NA	-	-	-	1 (1)	[28]
*A. lumbricoides*	Overall	1792	67 (61–72)	80.7	Asymmetry	0.5756	68 (63–73) (2 added studies)	9 (10)	[10,20,21,22,23,24,25,26,27]
	**Continent**								
	Africa	522	65 (57–73)	66.4	-	-	-	4 (4)	[20,21,23,24]
	South America	400	64 (55–72)	56.2	-	-	-	3 (3)	[22,26,27]
	Asia	558	68 (48–83)	95.4	-	-	-	1 (1)	[25]
	Mixed (multi-continent)	312	76 (70–80)	NA	-	-	-	1 (3)	[10]
	**Country**								
	Ethiopia	417	62 (54–69)	48.4	-	-	-	3 (3)	[20,23,24]
	Tanzania	105	74 (65–82)	NA	-	-	-	1 (1)	[21]
	Argentina	140	60 (52–68)	0	-	-	-	2 (2)	[22,26]
	Lao PDR	558	68 (48–83)	95.4	-	-	-	1 (2)	[25]
	Brazil	260	70 (64–75)	NA	-	-	-	1 (1)	[27]
	Mixed (multi-country)	312	76 (70–80)	NA	-	-	-	1 (3)	[10]
	**Participants**								
	Schoolchildren	1126	67 (61–73)	74.1	-	-	-	7 (7)	[10,20,21,22,23,24,27]
	Villagers	558	68 (48–83)	94.5	-	-	-	1 (2)	[25]
	Hospital patients/villagers	108	62 (52–71)	NA	-	-	-	1 (1)	[26]
*T. trichiura*	Overall	1253	79 (57–91)	96.9	NA	NA	NA	7 (8)	[10,20,21,23,24,25,27]
	**Continent**								
	Africa	739	71 (31–93)	98.2	-	-	-	4 (4)	[20,21,23,24]
	South America	15	47 (21–73)	NA	-	-	-	1 (1)	[27]
	Asia	154	94 (88–96)	0	-	-	-	1 (2)	[25]
	Mixed (multi-continent)	345	80 (76–84)	NA	-	-	-	1 (1)	[10]
	**Country**								
	Ethiopia	519	54 (21–84)	98	-	-	-	3 (3)	[20,23,24]
	Tanzania	220	95 (91–97)	NA	-	-	-	1 (1)	[21]
	Lao PDR	154	94 (88–96)	0	-	-	-	1 (2)	[25]
	Brazil	15	47 (21–73)	NA	-	-	-	1 (1)	[27]
	Mixed (multi-country)	345	80 (76–84)	NA	-	-	-	1 (1)	[10]
	**Participants**								
	Schoolchildren	1099	70 (42–88)	97.5	-	-	-	6 (6)	[10,20,21,23,24,27]
	Villagers	154	94 (88–96)	0	-	-	-	1 (2)	[25]
Hookworms	Overall	2104	71 (61–80)	89.9	Asymmetry	0.2298	67 (51–80) (1 added study)	9 (10)	[10,20,21,23,24,25,26,27,28]
	**Continent**								
	Africa	670	77 (49–90)	87.6	-	-	-	5 (5)	[20,21,23,24,28]
	South America	364	76 (65–85)	75.4	-	-	-	2 (2)	[26,27]
	Asia	780	62 (46–76)	94.8	-	-	-	1 (2)	[25]
	Mixed (multi-continent)	290	72 (67–77)	NA	-	-	-	1 (1)	[10]
	**Country**								
	Ethiopia	519	76 (43–93)	90.7	-	-	-	4 (4)	[20,23,24,28]
	Tanzania	151	68 (59–75)	NA	-	-	-	1 (1)	[21]
	Argentina	77	70 (59–80)	NA	-	-	-	1 (1)	[26]
	Lao PDR	780	62 (46–76)	94.8	-	-	-	1 (2)	[25]
	Brazil	287	81 (76–85)	NA	-	-	-	1 (1)	[27]
	Mixed (multi-country)	290	72 (67–77)	NA	-	-	-	1 (1)	[10]
	**Participants**								
	Schoolchildren	1001	76 (57–88)	90.1	-	-	-	6 (6)	[10,20,21,23,24,27]
	Villagers	780	62 (46–76)	94.8	-	-	-	1 (1)	[25]
	Hospital patients/villagers	77	70 (59–80)	NA	-	-	-	1 (1)	[26]
	Adult patients visiting health institutions	246	69 (63–74)	NA	-	-	-	1 (1)	[28]

NA: Not assessed; Subsets: authors using different flotation solutions for detecting parasites. Note: Differences in sample size across outcomes reflect outcome-specific data reported by the original studies.

## Data Availability

The original contributions presented in this study are included in the article/Supplementary Materials. Further inquiries can be directed to the corresponding author.

## Supplementary Materials

The following supporting information can be downloaded at https://www.mdpi.com/article/10.3390/tropicalmed11090249/s1. Table S1: Search terms; Table S2: Details of studies; Table S3: Methodological quality.
