# Supplementary material for: Diagnostic Sensitivity of the McMaster Technique for Detecting Intestinal Parasites in Human Populations: A Systematic Review and Meta-Analysis
Source: Trop Med Infect Dis. 2026 Sep 1;11(9):249. doi: 10.3390/tropicalmed11090249 (PMC13611625; doi:10.3390/tropicalmed11090249)
Supplement: Supplementary file 1 [file tropicalmed-11-00249-s001.zip › Table S1. Search terms.pdf]

Table S1. Search terms

General keywords

McMaster AND (parasite* OR parasitic) AND (human* OR patient*)

PubMed 30 January 2026

No.	Key concept	Search terms	Results
1.	McMaster	McMaster[MeSH Terms] OR McMaster[Text Word]	9,154
2.	Parasite	parasite*[MeSH Terms] OR parasitic[MeSH Terms] OR parasite*[Text Word]OR parasitic[Text Word]	273,482
	Human	human*[MeSH Terms] OR patient*[MeSH Terms] OR human*[Text Word] OR patient*[Text Word]	26,039,722
3.	1 AND 2 AND 3	#1 AND #2 AND #3	65

Scopus 30 January 2026

No.	Key concept	Search terms	Results
1.	McMaster	TITLE-ABS-KEY (McMaster)	14,422
2.	Parasite	TITLE-ABS-KEY (parasite* OR parasitic)	471,327
3.	Human	TITLE-ABS-KEY (human* OR patient*)	31,509,808
4.	1 AND 2 AND 3	(TITLE-ABS-KEY (McMaster)) AND (TITLE-ABS-KEY (parasite* OR parasitic)) AND (TITLE-ABS-KEY (human* OR patient*))	129

Ovid (Journal) 30 January 2026

No.	Key concept	Search terms	Results
1.	McMaster AND parasite AND Human	McMaster AND (parasite* OR parasitic) AND (human* OR patient*) limit to (ovid full text available and articles with abstracts and original articles)	313

Nursing & Allied Health Premium 30 January 2026

No.	Key concept	Search terms	Results
1.	McMaster AND parasite AND Human	McMaster AND (parasite* OR parasitic) AND (human* OR patient*)	795

Web of Science 30 January 2026

No.	Key concept	Search terms	Results

1.	McMaster AND parasite AND Human	McMaster AND (parasite* OR parasitic) AND (human* OR patient*)	212

Google Scholar 2 February 2026

No.	Key concept	Search terms	Results
1.	McMaster AND parasite AND Human	McMaster AND parasite AND Human	22,400 (Screening for the first 200 results)

Google Scholar (n = 200);

182 non-related articles

18 related articles were excluded with reasons;

- Animal samples (n=5)

- Protocol (n=2)

Final included (n = 11)

-Duplicated with articles selected from main databases (n = 6)

-Incomplete data (n = 5)
